# Life after loss: gendered changes in social networks and community participation around widowhood

**DOI:** 10.1093/geronb/gbag141

**Published:** 2026-07-23

**Authors:** Elisa Tambellini, Mirkka Danielsbacka, Antti O Tanskanen, Anna Rotkirch

**Affiliations:** Population Research Institute, Väestöliitto, Helsinki, Finland; INVEST Research Flagship Centre, University of Turku, Turku, Finland; Population Research Institute, Väestöliitto, Helsinki, Finland; INVEST Research Flagship Centre, University of Turku, Turku, Finland; Population Research Institute, Väestöliitto, Helsinki, Finland; Department of Social Research, University of Turku, Turku, Finland; Population Research Institute, Väestöliitto, Helsinki, Finland; (Social Sciences Section)

**Keywords:** Spousal loss, Social participation, Social ties, Longitudinal analysis, Later-life

## Abstract

**Objectives:**

This study examined how widowhood shapes older adults’ close network composition and quality and community participation, and whether these effects differ by gender.

**Methods:**

Data from the Survey of Health, Ageing and Retirement in Europe (SHARE) were used to analyze 55,555 Europeans aged 50 and older, including 3,257 who became widowed between survey waves. Fixed-effects panel models estimated within-person changes in network size, composition (number of friends, children, and others in the network), satisfaction with the network, emotional closeness (average level of closeness across all social network members), and community involvement (active involvement of individuals in collective and social activities) from before to after widowhood, with gender interactions testing for differential adaptation.

**Results:**

Widowhood was associated with sustained increases in network size and diversity, particularly in ties with children and friends, and especially among men. However, network satisfaction declined persistently following spousal loss for both genders, and emotional closeness deteriorated over time, though less so for women. Widowed women, more than men, increased their engagement in community activities, especially in voluntary work and sport or social clubs in the years following spousal loss.

**Discussion:**

Gendered patterns of social adaptation emerge after widowhood, with men showing larger increases in network size and in the presence of children, and women showing greater increases in community participation. Structural network growth after widowhood does not necessarily translate into improved relationship quality. Rather than women uniformly outperforming men in social adaptation, our results suggest that widowers and widows pursue distinct and complementary strategies.

The transition to widowhood is a profound and disruptive life event, reshaping not only personal identity but also social environments and daily routines (Lucas et al., 2003). For many older adults, a long-term partner represents the primary source of social support, emotional intimacy, and shared daily life, making spousal loss a transition with far-reaching consequences for health and well-being (Moon et al., 2011). Indeed, widowed individuals represent a particularly vulnerable group: bereavement is associated with elevated risks of depression, physical health decline, and mortality, especially in the period immediately following loss (Moon et al., 2011; Stroebe et al., 2007).

Social networks and community engagement play a central role in the transition to life after spousal loss. Research shows that structural support (including network size, contact frequency, and the presence of close confidants) as well as functional support (e.g., perceived emotional closeness and satisfaction with relationships), are key determinants of health adaptation and successful aging risk in later life (Holt-Lunstad, 2024; Rook & Charles, 2017). For widowed individuals specifically, support from children has been shown to buffer depression (Zheng & Yan, 2024), while perceived emotional support appears to mediate the relationship between bereavement-related anxiety and depression (Jacobson et al., 2017). Also, cultivating non-kin social ties in the wake of bereavement may be beneficial, in part because these ties help older adults maintain their independence and non-familial connectedness (e.g., Cornwell & Laumann, 2015).

Men and women enter widowhood with different social resources and participation patterns (Cohn-Schwartz & Schmitz, 2024; Cornwell, 2011). These pre-existing differences, combined with gender-specific coping strategies and recovery processes, may shape the social impact of widowhood. Research suggests that men are more prone to social isolation (Niino et al., 2025; Umberson et al., 2022) and may find it harder to establish new emotional supports following loss (Revenson et al., 1991).

This study analyzes longitudinal data from the Survey of Health, Ageing, and Retirement in Europe (SHARE) across 28 countries. We examine changes before and after widowhood in both structural and functional aspects of social life, including network characteristics and community participation. Specifically, we investigate three key research questions: *(1) How is widowhood associated with changes in the characteristics of close social networks, including size, composition, satisfaction, and emotional closeness to network members? (2) How is widowhood associated with a change in community involvement? (3) Do these effects differ between widowed men and women?*

Our research makes several contributions to the literature. First, we use a longitudinal fixed-effects design that enables within-person comparisons. Fixed-effects models address selection bias by using each individual as their own control, removing all stable, unobserved characteristics that might simultaneously predict both widowhood and social network outcomes, such as personality, early-life social skills, or genetic predispositions (Allison, 2009). Second, we adopt a multidimensional approach, examining both structural (network size and composition) and functional (satisfaction and closeness) aspects of social ties. Third, we incorporate community participation (e.g., organized activities, volunteering), providing a comprehensive view of social integration. Finally, by explicitly examining gender differences, we contribute to the understanding of how men and women adapt differently to a major life event and its social consequences.

## Theoretical background

### Social networks characteristics in later life and the social impact of widowhood

Social networks in later life provide essential resources for maintaining well-being and navigating life challenges. The convoy model conceptualizes these networks as protective layers of support that accompany individuals throughout life, adapting to changing circumstances and needs (Antonucci et al., 2014). Social network characteristics are well known to influence health and well-being outcomes in older adults (Litwin & Levinsky, 2023). Researchers commonly differentiate between structural dimensions (e.g., network size, contact frequency, relationship type) and functional dimensions (e.g., emotional and practical support received, perceived adequacy and quality), which interact to create unique configurations of social resources (August & Rook, 2020). Community participation, encompassing both formal activities (e.g., volunteering, religious attendance, organizational membership) and informal engagement (e.g., visiting friends, attending social events), involves a wider range of contacts than close networks and serves additional functions, including maintaining social roles and providing a sense of purpose (Greenfield & Marks, 2004).

Spousal death disrupts established social systems through multiple pathways: it eliminates a primary source of daily interaction and emotional support, forces a renegotiation of social roles and identities, and can trigger the loss of couple-based friendships (Martin-Matthews, 2011; Urbaniak et al., 2023). Several theories offer competing accounts of how widowhood shapes social life. Activity theory proposes that widowed individuals compensate for spousal loss by increasing social involvement and developing or deepening alternative ties (Cavan et al., 1949; Zettel & Rook, 2004). Disengagement theory predicts the opposite: a retreat from social life as a response to grief or broader age-related withdrawal (Zhang & Lin, 2022). Continuity theory suggests that pre-bereavement patterns of engagement largely persist (Atchley, 1989). More recent frameworks offer greater nuance: socioemotional selectivity theory (SST; Carstensen, 2021) predicts selective reorganization, a reduction of peripheral ties alongside deepening of emotionally significant ones, while hierarchical compensatory frameworks propose that support needs are redirected in a structured order from spouse to children, broader family, and friends (Zettel & Rook, 2004). Cornwell and Laumann (2018) further suggest that widowhood may paradoxically expand networks by forcing individuals into new roles and reactivating dormant ties. Finally, the dual process model (Stroebe & Schut, 1999) situates changes in social network within the broader bereavement process, framing the mobilization of non-spousal ties as part of restoration-oriented adaptation.

Empirical evidence regarding widowhood’s social effects presents a complex picture. Some studies document significant network contraction following spousal loss, particularly in the loss of couple-based friendships and reduced participation in shared activities (Donnelly & Hinterlong, 2010). Support for this view comes from research linking widowhood to changes in volunteering, social visits with family and friends, and religious engagement (Iveniuk et al., 2020; Lim-Soh, 2022). Other research suggests that while certain relationships may be lost, others may intensify, particularly those with adult children and long-term friends (Ha, 2008). The temporal dimension appears crucial: the immediate post-loss period tends to show the greatest disruption, followed by gradual adaptation and potential network reconstruction (Bennett 2005; Guiaux et al., 2007).

### Gender differences in social networks and widowhood adaptation

Gender significantly influences how people develop and maintain their social networks throughout their lives (Antonucci et al., 2014). Studies show that women typically have broader and more diverse social circles, often characterized by deeper emotional bonds (Haines & Hurlbert, 1992). Conversely, men’s social ties tend to be narrower, frequently revolving around their spouse, who usually acts as their primary source of emotional and social support (Cornwell, 2011). Moreover, within marriage, women often serve as the primary emotional caregivers, generally providing more consistent and higher-quality support than they receive (Neff & Karney, 2005). Therefore, the loss of a partner may have a more profound effect on men than on women (Stroebe & Stroebe, 1983). Women also frequently take on the role of maintaining social relationships within families—known as “kinkeeping”—which includes sustaining connections among extended kin (Gallagher & Gerstel, 1993). Consequently, widowers may find themselves with less support from their social circles, while widows are more apt to preserve and mobilize their existing relationships (Iveniuk et al., 2020).

These gender differences in social connections have important implications for how individuals respond to widowhood. Historically, much of the research on widowhood has focused primarily on women (e.g., Lopata, 1973), leading to a lack of studies examining gender-specific outcomes in the social domain. Nevertheless, existing findings suggest that widows may be better positioned to activate their social networks and seek help when needed, whereas widowers could be at greater risk due to having fewer social resources (Klaus, 2021). Previous studies show that widowed mothers are more likely to receive support from their children than widowed fathers (Kalmijn, 2007). Widowed mothers also report more frequent emotional and social contact with children, with widowed fathers being more likely than mothers to have no contact with at least one child (Jessee & Carr, 2025), a gap that is further pronounced when fathers have repartnered (Kalmijn, 2007). Additionally, they often report having larger social networks (Collins, 2018) and are more inclined to form new social ties compared to widowed men, who are more susceptible to social isolation and loneliness (e.g., Isherwood, 2023).

At the same time, widowed men are more likely than women to remarry (e.g., Schneider et al., 1996) and, despite smaller networks, may still engage in intergenerational support or volunteer activities, which serve as avenues for social engagement (Collins, 2018).

## Study aims

This study examines how spousal loss reshapes social ties and participation patterns, and whether these patterns differ by gender. Drawing on competing theoretical frameworks, we first formulated hypotheses related to structural dimensions:

**H1**. Activity theory predicts that widowed individuals will show larger close networks following spousal loss, as alternative relationships are activated or deepened.

Alternatively, in the case of H1, disengagement theory predicts that widowhood will be associated with smaller close networks and reduced diversity in tie composition, whereas continuity theory assumes that widowed individuals will show minimal change in network size and composition compared to pre-widowhood levels.

Beyond these directional predictions, SST and hierarchical compensatory frameworks converge on a more specific structural prediction:

**H2.** Following widowhood, close network composition will shift toward children and close friends, reflecting both motivated selectivity toward emotionally meaningful ties and compensatory substitution of the lost spousal role.

Next, hypotheses concerned the functional dimensions of the close social network. The dual process model suggests that:

**H3.** Widowed individuals will report maintained or improved satisfaction with their social relationships and emotional closeness to network members.

Then, we turned to community participation and study the following hypothesis based on disengagement theory:

**H4**. Widowhood is associated with reduced community participation across activity types.

In contrast, regarding H4, continuity theory assumes that widowed individuals will maintain pre-existing levels of community participation following spousal loss.

Extending the dual process model beyond close network ties, community participation may similarly serve as a vehicle for rebuilding social routines and roles, a form of adaptation that is also consistent with activity theory’s emphasis on compensatory engagement:

**H5.** Widowed individuals will increase their involvement in community participation following spousal loss.

Finally, we examined gender differences in adaptation patterns. Given well-documented evidence that widows are better positioned than widowers to activate existing social resources, we predict:

**H6.** Widowed women will show greater resilience than widowed men in maintaining network size, relationship quality, and community engagement.

## Data and sample

Data come from the SHARE, a longitudinal study examining health, socioeconomic conditions, and social relationships among individuals aged 50 and older across 28 countries (Börsch-Supan et al., 2013). The survey comprises nine waves of data collection conducted biennially from 2004 to 2022. For this analysis, we used data from Waves 1–8, with particular focus on Waves 4, 6, and 8, which included comprehensive social network modules capturing detailed information about participants’ social connections and community participation.

**Figure 1.**
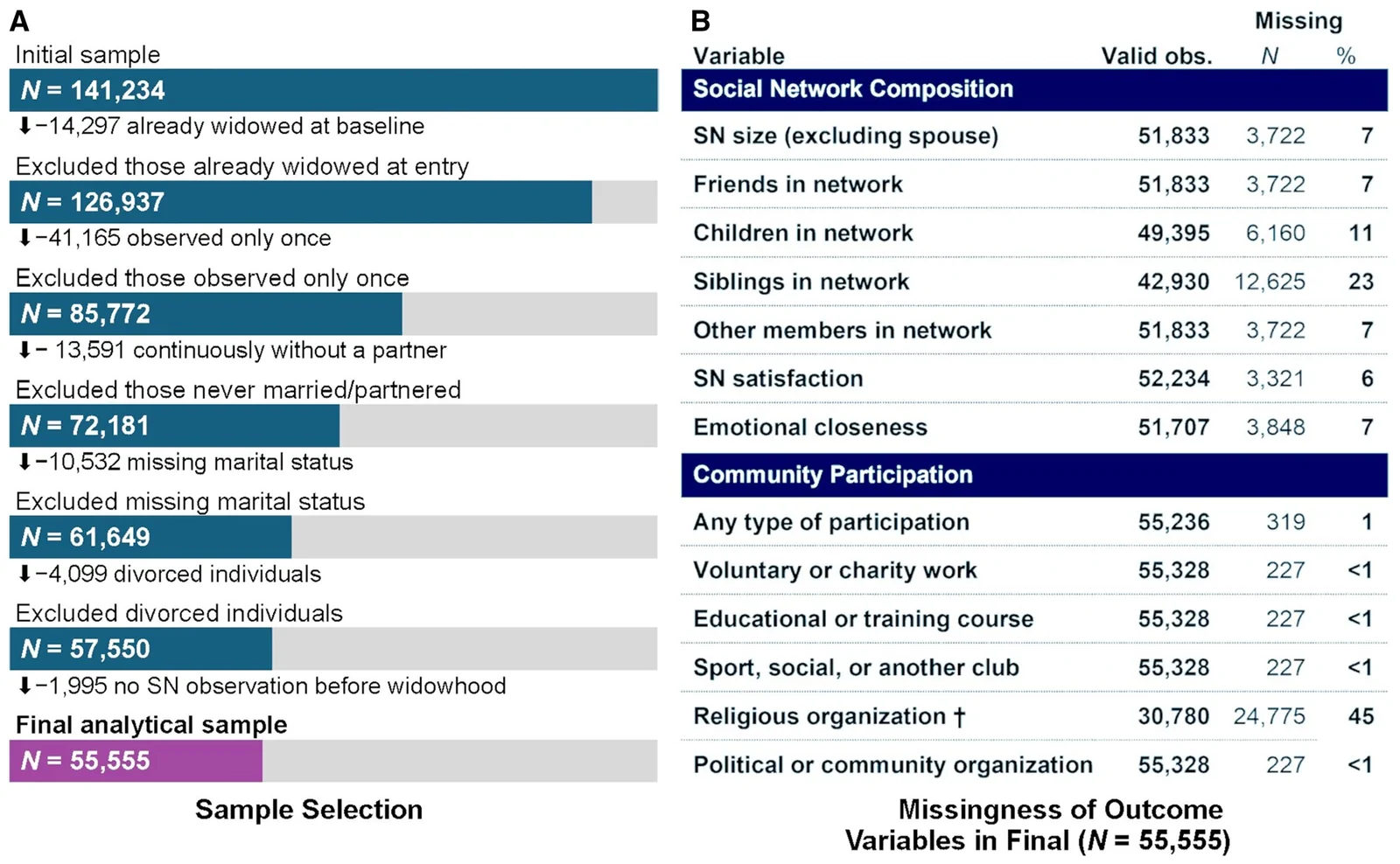
Sample selection process and missingness of study variables. (A) The sample selection process from the initial sample (*N* = 141,234) to the final analytical sample (*N* = 55,555). (B) Summary of the number and percentage of missing observations for social network composition, social network satisfaction and closeness, and community participation variables included in the final sample. SN = social network. ^†^High missingness for religious organization participation reflects that this item was not administered in all survey waves and countries.

To examine the effects of widowhood on social networks and participation patterns, our analytical approach required several sample restrictions, detailed in Figure 1 (Panel A). The initial sample included 141,234 individuals, we first excluded those already widowed at baseline (*n *= 14,297). We then excluded individuals observed only once (*n *= 41,165), and those who were continuously without a co-residing partner throughout the observation period (i.e., respondents for whom the data never records a partner present in the household; *n *= 13,591), those with missing partnership status information (*n *= 10,532), divorced individuals (*n *= 4,099), and those with no social network observation prior to widowhood (*n *= 1,995). The final analytical sample comprised 55,555 unique individuals from 28 countries (Austria, Belgium, Bulgaria, Croatia, Cyprus, Czech Republic, Denmark, Estonia, Finland, France, Germany, Greece, Hungary, Israel, Italy, Latvia, Lithuania, Luxembourg, Malta, Netherlands, Poland, Portugal, Romania, Slovakia, Slovenia, Spain, Sweden, and Switzerland), including those who experienced a widowhood transition during the observation period (3,257 individuals), alongside individuals who remained partnered throughout and serve as the comparison group.

The variation in sample sizes across analyses reflects differential missingness across outcome variables, as detailed in Figure 1 (Panel B). Missingness was low for most community participation variables; social network variables showed moderate missingness, with 7% missing for friends, other network members, and overall network size, 11% for children in the network, and 23% for siblings in the network. One participation variable (involvement in religious organizations) exhibited 45% missingness since this item was not administered across all survey waves and countries; it was therefore excluded from further analysis. We used all available data for each outcome, resulting in varying analytic sample sizes across models.

### Dependent variables

Social network changes were assessed across both structural and functional dimensions, following established theoretical frameworks emphasizing their distinct contributions to social integration and well-being (Cohen & Wills, 1985; Jackson & Antonucci, 1992). We also examined several specific community involvement activities, as well as a measure indicating whether respondents participated in at least one of them. The distribution of all these variables is shown in Table 1.

**Table 1:** Baseline characteristics of the analytical sample by gender and widowhood status.

Characteristics	Men		Women	
	Married	Widowed	Married	Widowed
***N***	26,435 (96.8%)	878 (3.2%)	25,863 (91.6%)	2,379 (8.4%)
**Sociodemographic**				
Age	63.86 (9.62)	70.49 (8.82)***	60.42 (9.39)	67.63 (8.43)***
Self-perceived health:				
Fair/poor	35.9%	44.6%***	35.1%	48.1%***
Retired	55.6%	79.9%***	38.1%	61.1%***
Life satisfaction	7.71 (1.75)	7.60 (1.81)	7.69 (1.77)	7.39 (1.86)***
**Social network**				
Spouse in network	91.3%	87.1%**	82.8%	74.8%***
Network size (excl. spouse)	1.43 (1.49)	1.40 (1.43)	1.97 (1.59)	1.94 (1.51)
Friends	0.29 (0.71)	0.25 (0.68)	0.43 (0.82)	0.34 (0.70)***
Children	0.74 (0.96)	0.84 (1.01)*	0.95 (0.98)	1.05 (0.97)***
Siblings	0.16 (0.47)	0.14 (0.40)	0.28 (0.57)	0.27 (0.59)
Others	0.10 (0.40)	0.08 (0.37)	0.11 (0.40)	0.11 (0.40)
Network satisfaction	8.88 (1.36)	8.94 (1.29)	8.97 (1.25)	9.00 (1.32)
Network closeness	3.32 (0.61)	3.21 (0.65)***	3.34 (0.58)	3.24 (0.59)***
**Community participation**				
**Any participation**	42.8%	37.4%**	41.9%	34.3%***
Voluntary/charity work	15.0%	13.4%	15.7%	10.3%***
Educational course	10.9%	5.2%***	14.8%	7.4%***
Sport or social club	27.5%	23.1%*	23.4%	18.3%***
Political/community org.	7.7%	6.5%	4.6%	2.5%***

*Notes*. Baseline refers to each respondent’s first observation in the survey, at which point all participants, including those who would later become widowed, were still married or partnered. Means (*SD*) reported for continuous variables; percentages for categorical variables. *t*-tests used for continuous variables, chi-square tests for categorical variables. Frequencies omitted for brevity; full counts available upon request.

* *p* < .05,

** *p* < .01,

*** *p* < .001.

Structural components of the social network included both its size and composition. Network size refers to the total number of close social connections and is assessed using SHARE’s ego-centered approach. Respondents were asked to list up to seven individuals with whom they frequently discussed important matters in the past 12 months. The resulting measure ranged from 0 to 7, with the spouse or partner excluded from the count to focus on non-spousal social resources.

Network composition captured the diversity of relationship types within a respondent’s close network. Specifically, it reflected the number of distinct categories of ties. The SHARE module categorizes each nominated person as a spouse/partner, parent, in-law, sibling, child, extended relative, or non-relative. For our analysis, we focused on counts of friends, siblings, children, and other non-relatives and non-friends (e.g., neighbors or ex-colleagues). The resulting measures also ranged from 0 to 7.

Functional components addressed perceived social support, including the quality of the network and emotional closeness among members. This dimension reflected the depth and significance of social ties, such as the strength of emotional bonds within the network (Antonucci, 2001). Satisfaction with the social network was measured using the question: “Overall, on a scale from 0 to 10, where 0 means completely dissatisfied and 10 means completely satisfied, how satisfied are you with the relationship you have with the person/relationships you have with all the people we just discussed?.” Finally, emotional closeness was assessed using the question: “How close do you feel to the person/relationships you have with all the people we just discussed?” The response options are as follows: 1) *Not very close*, 2) *Somewhat close*, 3) *Very close*, and 4) *Extremely close*. Based on the responses provided for each person mentioned, an average closeness variable was constructed, ranging from 1 (*not very close*) to 4 (*extremely close*). All social network variables were standardized (mean = 0, *SD* = 1) to ensure direct comparability of coefficients across models in the subsequent analyses.

Participation in social or community activities was based on the SHARE question: “Which of the activities listed on this card—if any—have you done in the last twelve months?.” Respondents were considered socially active if they reported engaging in at least one of the following activities: voluntary or charity work; attending an educational or training course; going to a sport, social, or other club; or taking part in a political or community-related organization. Each participation measure was coded as a binary variable (participated vs. did not participate), and a binary measure was created to indicate whether respondents participated in at least one of them.

### Independent variables

The independent variable captured temporal proximity to spousal loss. Partnership status was operationalized using a combination of the SHARE household-grid variable and self-reported marital status. Respondents were defined as partnered when the household-grid variable indicated a partner was present in the household and their self-reported marital status was married, registered partnership, or never married. This approach captured de facto co-residential partnerships across different legal statuses while excluding individuals who report being divorced or separated. Widowhood status was determined from respondents’ self-reported relationship status, with individuals classified as widowed if they selected this option from available categories (married, registered partnership, never married, divorced/separated, widowed), conditional on a non-missing value in the variable recording the year of the partner’s death.

Recognizing that major life events involve both anticipation and adaptation processes (Frijters et al., 2011), we constructed a time-since-widowhood variable. Specifically, we used the interview year and the reported calendar year of the spouse’s death to calculate the number of years elapsed since spousal loss as the difference between the two. Based on this year-level measure, we constructed four dummy indicators reflecting distinct phases relative to widowhood: 2 or more years before spousal loss (*t* ≤ −2), 1 year before (*t* = −1), the year of widowhood (*t* = 0), 1 year after (*t* = 1), and 2 or more years after (*t* ≥ 2).

### Control variables

Gender was coded as a binary variable (male/female) based on self-reports. It was included as the primary moderator, interacting with time since widowhood, to assess whether the effects of widowhood differ between men and women.

Sociodemographic factors were controlled for in the data analysis, including age (in years), employment status (not retired/retired), life satisfaction, and overall health status. The distribution of these variables at the sample baseline is shown in Table 1.

Self-rated health was measured on a five-point scale (*excellent*, *very good*, *good*, *fair*, *poor*) and recoded into a binary indicator. Respondents reporting *excellent*, *very good*, or *good health* were classified as being in good health (coded 1), while those reporting fair or poor health were classified as being in *fair*/*poor health* (coded 0). Life satisfaction was assessed with the question “On a scale from 0 to 10, where 0 means completely dissatisfied and 10 means completely satisfied, how satisfied are you with your life?” Higher scores indicate *greater life satisfaction*.

### Analytical strategy

Fixed-effects panel regression models were deployed to estimate within-individual changes in social network characteristics and community participation in response to widowhood. This approach controls for all time-invariant individual characteristics, both observed and unobserved, by focusing on changes within the same person over time (Brüderl & Ludwig, 2015). The baseline model takes the following form:


$$Y_{it}=\alpha_{i}+\Sigma_{k}\beta_{k}D_{it}^{k}+\gamma X_{it}+\varepsilon_{it}$$

where *Y_it_* is the outcome for individual *i* at time *t*; *α_i_* is an individual fixed effect capturing all time-invariant characteristics; $D_{\mathrm{it}}^{k}$ are dummy indicators for widowhood timing, with *k* indexing five categories: 2 or more years before spousal loss (*t* ≤ −2, the reference category), 1 year before (*t *= −1), the year of widowhood (*t *= 0), 1 year after (*t *= 1), and 2 or more years after (*t *≥ 2); *X_it_* is a vector of time-varying controls including age, self-rated health, retirement status, and life satisfaction; and ε_*it*_ is the idiosyncratic error term.

To examine gender heterogeneity, we extended the baseline model by adding interactions between the widowhood-timing indicators and a female indicator:


$$Y_{it}=\alpha_{i}+\Sigma_{k}\beta_{k}D_{it}^{k}+\Sigma_{k}\delta_{k}(D_{it}^{k}\times {\mathrm{Female}}_{i})+\gamma X_{it}+\varepsilon_{it}$$

where *δ_k_* captures the differential effect of widowhood timing for women relative to men. Since gender was time-invariant, it was absorbed by the individual fixed effect *α_i_* and does not appear as a main effect in the model.

For outcomes measuring social network characteristics (network size, number of different network member types, network satisfaction, and emotional closeness), we estimated linear fixed-effects models. To facilitate comparability of effect sizes across outcomes, all network variables were standardized prior to estimation.

For community participation outcomes, although these were binary indicators, we estimated linear probability models with individual fixed effects rather than fixed-effects logit models. Linear fixed-effects models generally retain groups even when the outcome variable does not vary within groups, because OLS estimation absorbs group-level fixed effects through within-group demeaning rather than conditioning on outcome variation. By contrast, fixed-effects logit models rely on conditional maximum likelihood estimation, which conditions on the within-group sum of the outcome variable. Groups in which the outcome does not vary contribute no information to the conditional likelihood and are therefore automatically dropped from estimation (Chamberlain, 1980). The linear probability model avoids this issue; moreover, they provide consistent estimates of average marginal effects expressed as percentage-point changes, and is well-suited to large samples (Angrist & Pischke, 2009). For both sets of models, standard errors were clustered at the individual level.

As a robustness check for the social network models, we re-estimated all specifications using binary versions of the network composition and satisfaction variable. Specifically, network composition outcomes were recoded as indicators of whether the respondent had any contact of a given type (i.e., any friend, any child, any sibling, any other network member), and network satisfaction was dichotomized as low versus high satisfaction (below 8 versus 8 or above). Results from these robustness checks are reported in Supplementary Table 2) and are broadly consistent with the main findings.

## Results

### Sample characteristics

Table 1 presents the baseline characteristics of the analytical sample separately by gender and widowhood status. Baseline refers to each respondent’s first observation in the survey, at which point all participants were still married or partnered. Individuals are grouped according to whether they subsequently became widowed during the observation period or remained continuously partnered. Among men, 878 (3.2%) experienced widowhood. Among women, the proportion experiencing widowhood was substantially higher, with 2,379 women (8.4%) becoming widowed. Widowed individuals of both genders were considerably older than their continuously married counterparts, and more likely to report less fair/poor health. Widowed respondents were also substantially more likely to be retired, particularly among men (79.9% vs. 55.6%).

Regarding social networks, widowed respondents of both genders showed lower network closeness compared to married respondents, while network size and network satisfaction remained broadly similar across groups. Notably, widowed women reported fewer friends in their network than their married counterparts (0.34 vs. 0.43), while widowed men and women both reported slightly more children in their network.

Finally, community participation was lower among widowed respondents across most activity types. The gap was particularly pronounced for educational courses, with widowed men and women participating at roughly half the rate of their married counterparts, and was statistically significant for most activity types among women, though less consistently so among men.

### Social network structure

**Figure 2:**
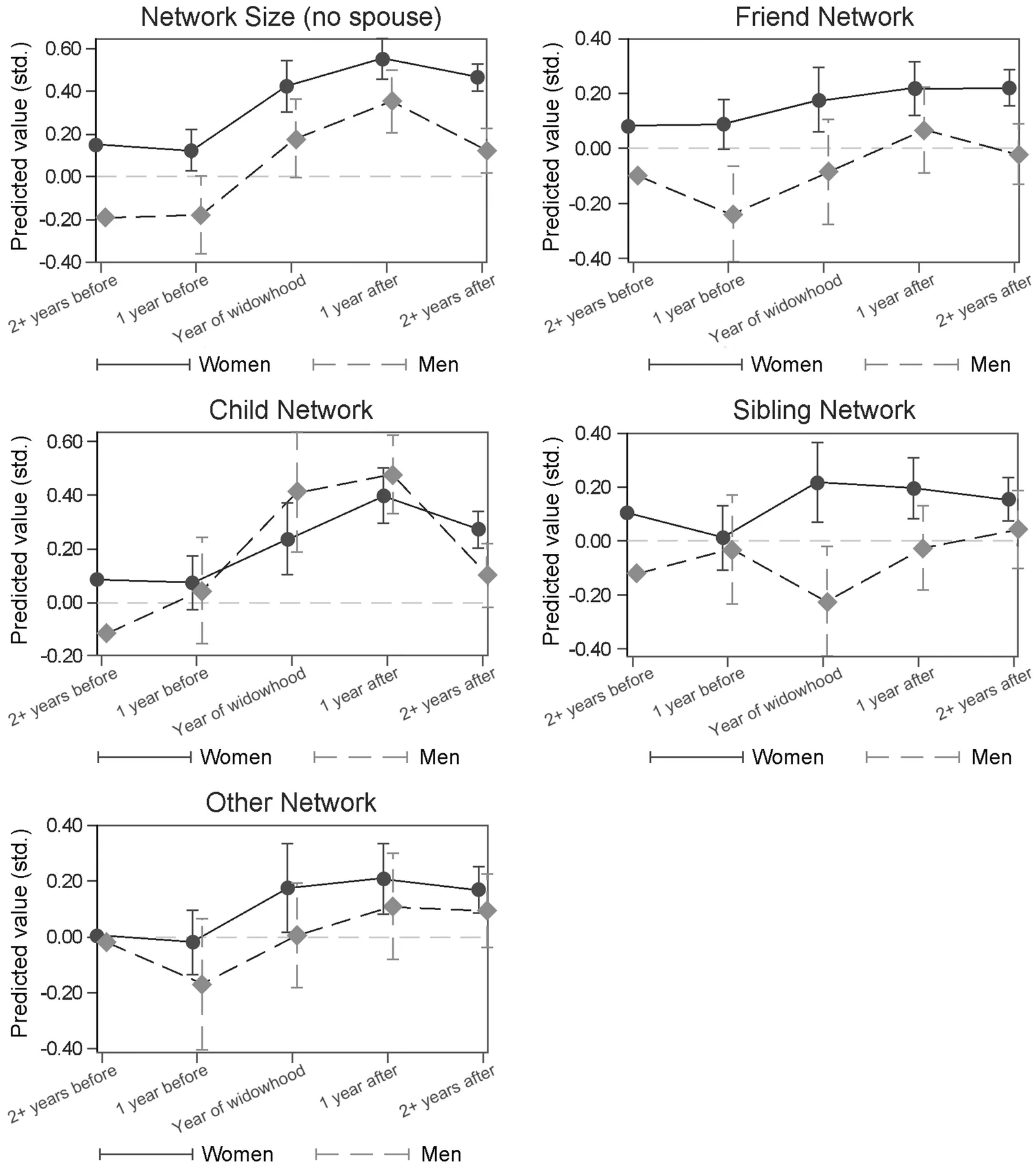
Network size and composition around widowhood: predicted margins by gender. *Note*. Predicted margins were estimated from fixed-effects models. Error bars indicate 95% confidence intervals. Controls: age, self-rated health, retirement status, life satisfaction.

Figure 2 displays predicted margins from fixed-effects models for network size and composition across widowhood periods, separately by gender. Full regression estimates, including gender interaction terms, are reported in Supplementary Table 3.

Network size (excluding the spouse) increased significantly following spousal loss. Relative to the reference period (more than 2 years before widowhood), the increase became statistically significant from the year of widowhood onwards and remained sustained through 2 years or more after (Supplementary Table 3, Model 1a). This pattern is consistent with H1 as formulated by activity theory. However, the trajectory differed by gender, providing partial support for H6. Women entered widowhood with larger networks and maintained higher absolute predicted values throughout (Figure 2), but men exhibited a steeper post-widowhood increase: the negative and significant interaction term after 1 year or more (−0.18, *p* < .05, Supplementary Table 3, Model 1 b) indicates that women’s gain relative to the pre-widowhood baseline was significantly smaller than men’s.

Turning to network composition, the results provide clear support for H2. The number of children in the network showed the largest and most consistent post-widowhood expansion, with large and highly significant main effects across all post-widowhood periods (e.g., 1+ years after: 0.39***, Supplementary Table 3, Model 3a), alongside a significant increase in friends in the networks (1+ years after: 0.148***, Supplementary Table 3, Model 2a).

Gender differences in composition do not support H6. The number of children in the network expansion was significantly stronger for men, as indicated by negative and significant interactions at the year of widowhood (−0.41***) and 1 year and more (−0.32***). As Figure 1 illustrates, women maintained higher absolute levels of child contact throughout, reflecting stronger pre-existing ties, while men showed a steeper post-widowhood increase, consistent with a catching-up process among those with lower baseline investment in kin ties (Supplementary Table 3, Models 3a and b; Figure 2). Number of friends in the network, by contrast, showed no significant gender interactions, indicating similar rates of change for both genders despite women’s higher baseline levels. Sibling and other networks showed more modest and less consistent effects (Supplementary Table 3, Models 4a and 5a), but with some evidence of gender differences (Supplementary Table 3, Model 4b) in the number of siblings.

### Social network quality

**Figure 3:**
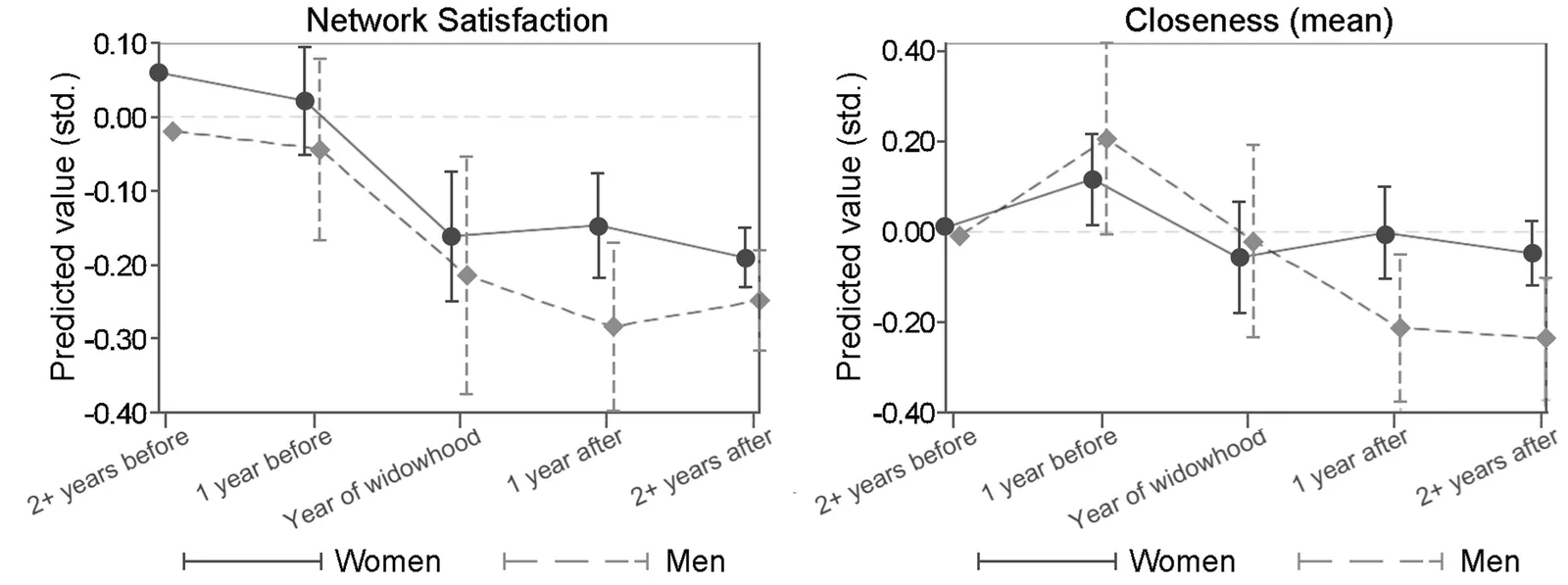
Network quality around widowhood: predicted margins by gender. *Note*. Predicted margins were estimated from fixed-effects models. Error bars indicate 95% confidence intervals. Controls: age, self-rated health, retirement status, life satisfaction.

Figure 3 shows predicted margins for network satisfaction and emotional closeness. Corresponding estimates are reported in Supplementary Table 3 (Models 6a and b and 7a and b). The results do not support H3. Rather than maintaining or improving satisfaction and closeness following spousal loss, widowed individuals experienced a sharp and persistent decline in network satisfaction. The effect emerged from the year of widowhood (−0.21**, Supplementary Table 3, Model 6 b) and remains stable through 2 and more years after (−0.25***). This deterioration in perceived quality occurred simultaneously with the structural expansion documented above, indicating that network growth does not translate into improved relationship quality. Critically, none of the gender interaction terms for satisfaction were statistically significant, meaning this decline was equally pronounced for men and women, providing no support for H6 on this dimension.

Emotional closeness showed a more nuanced pattern. In the pooled model, closeness actually increased slightly in the year before widowhood (0.13***, Supplementary Table 3, Model 7a), possibly reflecting anticipatory mobilization of emotional bonds, before declining significantly in the longer run (2+ years after: −0.09***), again inconsistent with H3. However, a significant gender difference emerged here in partial support of H6: the positive and significant interactions at 1 year or more and 2 years or more after (0.21** and 0.20** respectively, Supplementary Table 3, Model 7 b) indicate that men experienced a steeper long-run decline in emotional closeness than women. Figure 3 illustrates this divergence, with men’s predicted closeness falling more sharply over time while women’s trajectory remains more stable.

### Community participation

**Figure 4:**
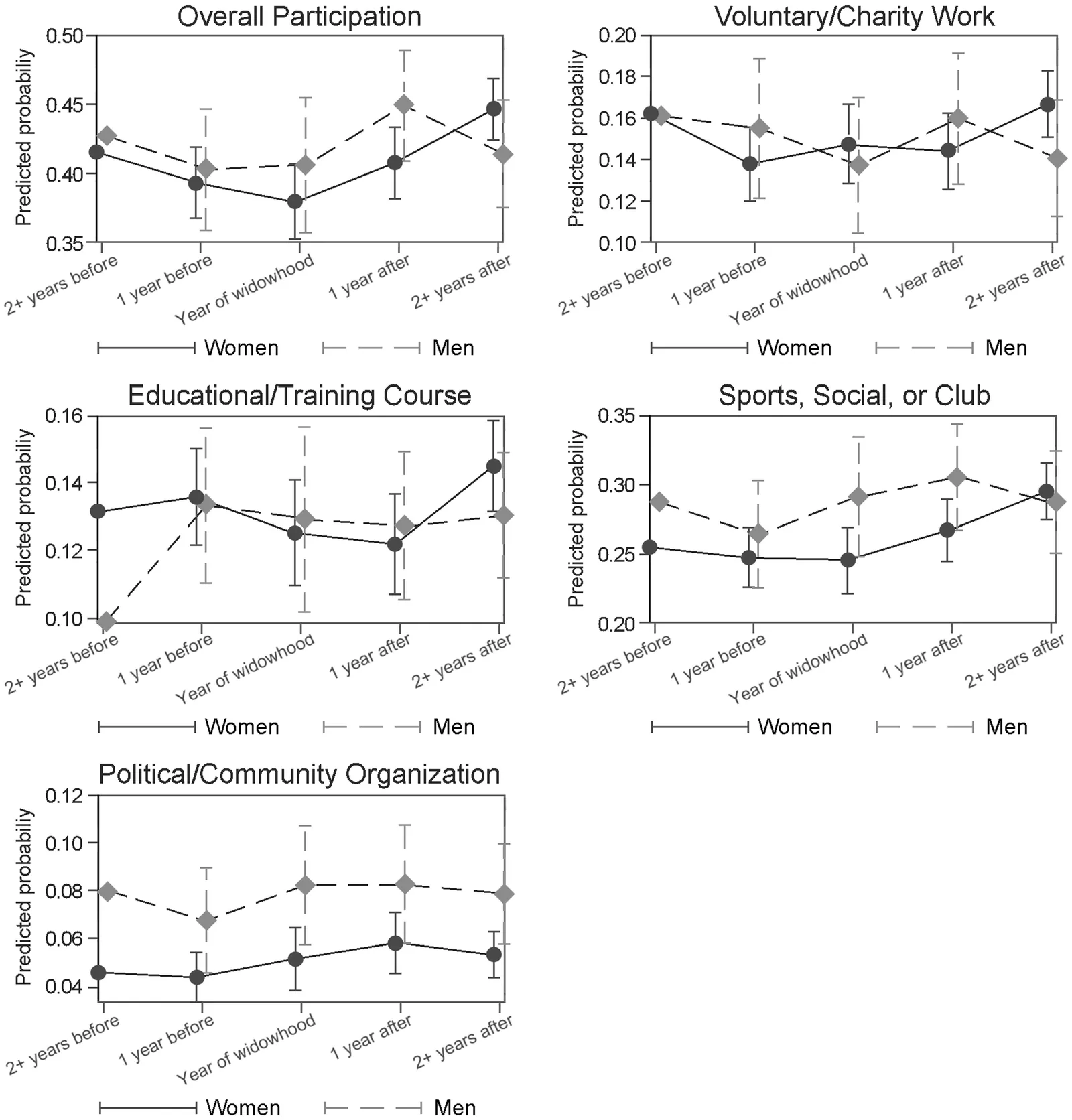
Community participation around widowhood: predicted probabilities by gender. *Note*. Predicted probabilities were estimated from fixed-effects models. Error bars indicate 95% confidence intervals. Controls: age, self-rated health, retirement status, life satisfaction.

Figure 4 displays predicted probabilities of participation across widowhood periods by gender, with full estimates reported in Supplementary Table 4. Overall participation levels remained stable around widowhood for both genders (Figure 4, Supplementary Table 4, Models 1a and b), with predicted probabilities hovering between 0.40 and 0.45 throughout. This pattern is broadly consistent with continuity theory’s expectation of maintained participation and provides no support for the disengagement theory prediction underlying H4 of reduced community involvement. At the same time, evidence in favor of H5 (increased participation following spousal loss) emerged primarily in the longer term.

At the activity level, voluntary and charity work showed a modest initial decline around widowhood for both genders, followed by a recovery that is significantly stronger for women in the longer term (interaction at 2+ years after: 0.03**, Supplementary Table 4, Model 4b). A parallel pattern was observed for sport, social, or club participation, where women’s predicted probability converges toward and ultimately exceeds men’s (interaction at 2+ years after: 0.08***, Supplementary Table 4, Model 8). These findings lend support to H5 specifically for women and in the post-acute phase of bereavement, as anticipated by both activity theory and an extension of the dual process model to community engagement.

Educational and training course participation showed the opposite gender pattern, which is inconsistent with H6. Men exhibited an increasing trend in educational engagement post-widowhood (Figure 4), while women did not follow the same trajectory. Significant negative interactions in the year of widowhood and 1 year and more after (both −0.03**, Supplementary Table 4, Model 6b) confirm this pattern.

Political and community organization participation showed no significant change around widowhood for either gender, and no significant interactions emerged (Supplementary Table 4, Models 9 and 10), providing neither support for H4 nor H5 on this dimension. The pre-existing gap, with men maintaining approximately twice the predicted probability of women, remained stable throughout the observation window (Figure 4).

## Discussion and conclusion

This longitudinal study shows that widowhood in contemporary Europe prompts distinct patterns of social network restructuring and community engagement, with gender differences in community involvement that persist over time. Fixed-effects models on SHARE panel data across 27 European countries and Israel showed that spousal loss leads to sustained expansion of social networks, with widowed individuals increasing their overall network size immediately following spousal loss and maintaining these larger networks over subsequent years. This expansion occurs primarily through strengthened connections with children and friends.

Our results are consistent with activity theory’s emphasis on adaptation and role substitution following major life transitions (Cavan et al., 1949) and align with Cornwell and Laumann’s (2018) observation that widowhood can expand social networks by pushing individuals into new roles and reactivating dormant ties. Our findings also extend those of Guiaux et al. (2007), who found evidence of gradual network reconstruction following an initial period of disruption, by showing that expansion is not only sustained but continues across multiple waves.

A critical and somewhat paradoxical finding concerns the disconnect between network structure and quality. While social ties expand quantitatively, emotional closeness significantly decreases in the longer term, and network satisfaction declines persistently following spousal loss. These findings also invite a direct engagement with SST (Carstensen, 2021), which predicts that as individuals perceive their time horizon as shrinking, they increasingly prioritize emotionally close and meaningful relationships over the expansion of their social world. Our findings stand in tension with this prediction. One interpretation is that widowhood disrupts the selectivity process itself: rather than being able to concentrate on existing high-quality ties, bereaved individuals enter a period of active network reconstruction that prioritizes breadth, at least in the short- to medium-term. This pattern resonates with Ha’s (2008) distinction between the availability of social ties and their perceived quality and underscores the irreplaceable nature of the spousal bond as a source of deep emotional intimacy.

The gender findings reveal a nuanced picture. Our results suggest that widowers and widows pursue distinct strategies shaped by pre-existing differences in network structure and social roles. A key insight concerns the number of children in the network. Women maintain stronger ties with children both before and after spousal loss, while men show a steeper increase following bereavement. This pattern closely mirrors findings from SHARE data on grandparental childcare, where the transition to retirement produced a stronger increase in care provided by grandfathers than grandmothers, even though grandmothers provided more care both before and after retirement (Tanskanen et al., 2021). The same logic applies here: because women may already occupy a central role in family networks and maintain closer ties to children prior to widowhood, there is less room for further expansion. Men, by contrast, enter widowhood with lower baseline levels of child contact and may be more strongly motivated to compensate for the loss of their primary social anchor through intensified ties with children (Cornwell, 2011; Stroebe & Stroebe, 1983).

A parallel argument applies to community participation. Women’s broader pre-existing social capital (Collins, 2018; Gallagher & Gerstel, 1993) provides the social infrastructure needed to translate bereavement into outward community engagement, explaining their greater and more sustained increases in voluntary work and social club participation in the longer term. Men, instead, concentrate their adaptive efforts on close network reconstruction rather than broader community reintegration. Women’s relative advantage in emotional closeness over time further refines this picture: while men expand their networks more rapidly in structural terms, women appear more effective at maintaining the quality of those ties.

Moreover, an important contextual factor is the gendered patterns of caregiving. Women are more likely to have served as primary caregivers to their spouses prior to bereavement (e.g., DiGiacomo et al., 2013), a period during which social engagement often contracts. For them in particular, the post-widowhood increase in community participation may reflect a release from caregiving demands as much as a response to spousal loss per se. Future research would be needed to disentangle these pathways, and we acknowledge the absence of such measures as a limitation of the current study. We also acknowledge that some gender-specific interaction effects may be underpowered in smaller subgroups, particularly for less common activity types. However, the overall pattern of results is coherent across multiple outcomes and specifications, suggesting that these findings reflect substantive gender differences in adaptation rather than statistical artifacts.

Other limitations should be recognized. We retain all individuals with a co-residing partner as the reference category, on the grounds that they share the key structural feature of our analysis: the presence of a partner in the household and thus exposure to the risk of partner loss. However, this group is heterogeneous with respect to partnership type, encompassing legally married couples, registered partnerships, and informal cohabiting arrangements. To the extent that the emotional, social, and material consequences of bereavement differ across partnership types, our estimates may conflate effects that vary systematically across these subgroups. Moreover, by focusing on the transition from partnered to widowed, our study cannot fully disentangle the emotional consequences of bereavement from the structural consequences of partnership dissolution more broadly. Divorce also entails the loss of a co-residing partner and a reorganization of household and social life, but without the experience of bereavement. A comparison between widowed and divorced individuals could, in principle, help clarify the extent to which the patterns we document reflect grief and emotional loss specifically, or partner loss in any form. However, divorce transitions are rare in our sample, precluding a meaningful fixed-effects analysis of this group. We flag this as an important direction for future research.

A further limitation concerns the selectivity of our analytical sample. Because the fixed-effects approach requires observing individuals before and after widowhood, those already widowed at first survey entry are excluded. This group may differ from newly widowed individuals, as they have had more time to adjust and reconstruct their social networks. Descriptive statistics in Supplementary Table 5 suggest that already-widowed individuals have larger networks and more children in their networks than newly widowed individuals, consistent with ongoing network reconstruction over time. However, they also report slightly lower network satisfaction and emotional closeness than partnered individuals, indicating that some emotional consequences of spousal loss may persist long term. These findings should be interpreted cautiously, as they are based on cross-sectional descriptives that may reflect pre-existing differences and selective survival.

A further limitation concerns the potential gender bias embedded in the community participation measures used in this study. Focusing primarily on public-facing and formally organized activities risks underestimating women’s social engagement, which may be more strongly expressed through informal and private channels. We have sought to mitigate this by combining formal dimensions of participation with informal network measures capturing ties with friends and family. Future research would benefit from more comprehensive and gender-sensitive measures of social participation.

Finally, the use of SHARE data across 27 European countries and Israel offers considerable analytical breadth but also introduces important heterogeneity that warrants discussion. These countries vary substantially in their welfare state structures, cultural norms around gender roles, availability of formal social services, and the extent to which community participation is institutionally supported (Lakomý, 2021). These differences may moderate the effects of widowhood on social networks and participation in ways our pooled analyses cannot fully capture. We encourage future research to examine cross-national variation in widowhood’s social effects more explicitly, as this could illuminate how institutional and cultural contexts shape the pathways through which older adults rebuild their social worlds.

Despite these limitations, the findings carry important implications for supporting widowed individuals, with differences for men and women. While women appear to gravitate toward community-based structured activities, men may benefit from targeted pathways to social engagement that align with their existing social preferences and patterns. Crucially, given that network expansion does not automatically translate to increased emotional closeness, interventions should focus not only on facilitating new connections but on supporting the development of meaningful, emotionally supportive relationships that can partially compensate for the profound loss of the spousal bond.

## Supplementary Material

gbag141_Supplementary_Data

## Data Availability

The analyses are based on data from the Survey of Health, Ageing and Retirement in Europe (SHARE). SHARE data are not publicly downloadable but are available free of charge to registered researchers for scientific use. Data access can be requested through the SHARE Research Data Center (www.share-project.org), where users must complete a short application and agree to the terms of use. No new data were generated for this study.
