# Supplementary material for: Life after loss: gendered changes in social networks and community participation around widowhood
Source: J Gerontol B Psychol Sci Soc Sci. 2026 Jul 23;81(10):gbag141. doi: 10.1093/geronb/gbag141 (PMC13553083; doi:10.1093/geronb/gbag141)
Supplement: Supplementary file 1 [file gbag141_supplementary_data.docx]

***The Journals of Gerontology, Series B: Psychological Sciences and Social Sciences* Supplementary Material: Tambellini et al. Life after loss: Gendered changes in social networks and community participation around widowhood.**

**Supplementary Table 1.** Widowhood category and gender

|  | **Male** | **Female** |
| --- | --- | --- |
| Widowhood timing |  |  |
| 2+ year before | 1,737 (45.9%) | 4,490 (44.0%) |
| 1 year before | 410 (10.8%) | 1,157 (11.3%) |
| Year of widowhood | 283 (7.5%) | 787 (7.7%) |
| 1+ years after | 453 (12.0%) | 1,199 (11.8%) |
| 2+ years after | 902 (23.8%) | 2,563 (25.1%) |
| Obs. | 3,785 (27.1%) | 10,196 (72.9%) |
| *N* | 878 | 2,379 |

**Supplementary Table 2.** Widowhood Timing and Gender Effects on Social Network Characteristics: Binary Outcomes, Models (b) include Gender Interactions

|  | **(1a)** | **(1b)** | **(2a)** | **(2b)** | **(3a)** | **(3b)** | **(4a)** | **(4b)** | **(5a)** | **(5b)** |
| --- | --- | --- | --- | --- | --- | --- | --- | --- | --- | --- |
|  | **Any friend** | | **Any child** | | **Any sibling** | | **Any other** | | **Satisfaction** | |
| Widowhood period: 2+ years before (ref.) | | | | | | | | | | |
| 1 year before | 0.01 | -0.04 | -0.01 | 0.08^*^ | 0.00 | 0.04 | -0.02 | -0.04 | -0.00 | 0.03 |
| Year of widowhood | 0.05^**^ | 0.01 | 0.12^***^ | 0.26^***^ | 0.01 | -0.04 | 0.04^**^ | 0.00 | -0.00 | 0.03 |
| 1+ years after | 0.04^**^ | 0.03 | 0.18^***^ | 0.31^***^ | 0.03 | 0.03 | 0.06^***^ | 0.02 | 0.01 | -0.01 |
| 2+ years after | 0.06^***^ | 0.07^***^ | 0.09^***^ | 0.16^***^ | 0.03^*^ | 0.07^**^ | 0.05^***^ | 0.04^*^ | -0.01 | -0.02 |
| Period*gender |  |  |  |  |  |  |  |  |  |  |
| 1 year before # Female |  | 0.05 |  | -0.11^**^ |  | -0.05 |  | 0.03 |  | -0.04 |
| Year of widowhood # Female |  | 0.06 |  | -0.18^***^ |  | 0.07 |  | 0.05 |  | -0.04 |
| 1+ years after # Female |  | 0.01 |  | -0.19^***^ |  | -0.01 |  | 0.05^*^ |  | 0.04 |
| 2+ years after # Female |  | -0.00 |  | -0.10^***^ |  | -0.05 |  | 0.01 |  | 0.01 |
| Observations | 88,969 | 88,969 | 84,479 | 84,479 | 72,306 | 72,306 | 88,969 | 88,969 | 90,176 | 90,176 |

^*^ *p* < 0.10, ^**^ *p* < 0.05, ^***^ *p* < 0.01

**Supplementary Table 3.** Widowhood Timing and Gender Effects on Social Network Composition and Quality: Standardized outcomes variables (Models B Include Gender Interactions)

|  | **(1a)** | **(1b)** | **(2a)** | **(2b)** | **(3a)** | **(3b)** | **(4a)** | **(4b)** | **(5a)** | **(5b)** | **(6a)** | **(6b)** | **(7a)** | **(7b)** |
| --- | --- | --- | --- | --- | --- | --- | --- | --- | --- | --- | --- | --- | --- | --- |
|  | **SN size** | | **Friends** | | **Children** | | **Siblings** | | **Other** | | **Satisfaction** | | **Closeness** | |
| Widowhood period: 2+ years before (ref.) | | | | | | | | | | | | | | |
| 1 year before | -0.02 | 0.02 | -0.0262 | -0.15 | 0.019 | 0.16 | -0.05 | 0.09 | -0.06 | -0.15 | -0.03 | -0.03 | 0.13^***^ | 0.21^*^ |
| Year of widowhood | 0.29^***^ | 0.39^***^ | 0.0787 | 0.01 | 0.23^***^ | 0.54^***^ | 0.05 | -0.09 | 0.13^**^ | 0.02 | -0.21^***^ | -0.21^**^ | -0.05 | -0.02 |
| 1+ years after | 0.44^***^ | 0.56^***^ | 0.148^***^ | 0.15^**^ | 0.39^***^ | 0.61^***^ | 0.09^*^ | 0.11 | 0.18^***^ | 0.12 | -0.20^***^ | -0.28^***^ | -0.07 | -0.21^***^ |
| 2+ years after | 0.30^***^ | 0.34^***^ | 0.127^***^ | 0.06 | 0.18^***^ | 0.24^***^ | 0.07^*^ | 0.18^**^ | 0.15^***^ | 0.10 | -0.23^***^ | -0.25^***^ | -0.09^***^ | -0.24^***^ |
| Period*gender |  |  |  |  |  |  |  |  |  |  |  |  |  |  |
| 1 year before # Female |  | -0.06 |  | 0.16 |  | -0.19 |  | -0.19 |  | 0.13 |  | 0.00 |  | -0.10 |
| Year of widowhood # Female |  | -0.13 |  | 0.10 |  | -0.41^***^ |  | 0.19 |  | 0.15 |  | 0.00 |  | -0.04 |
| 1+ years after # Female |  | -0.18^**^ |  | -0.01 |  | -0.32^***^ |  | -0.03 |  | 0.08 |  | 0.09 |  | 0.21^**^ |
| 2+ years after # Female |  | -0.05 |  | 0.08 |  | -0.08 |  | -0.15^*^ |  | 0.06 |  | 0.03 |  | 0.20^**^ |
| Observations | 88,969 | 88,969 | 88,969 | 88,969 | 84,479 | 84,479 | 72,306 | 72,306 | 88,969 | 88,969 | 94,862 | 94,862 | 88,556 | 88,556 |

^*^ *p* < 0.10, ^**^ *p* < 0.05, ^***^ *p* < 0.01

**Supplementary Table 4.** Widowhood Timing and Gender Effects on Community Participation. Models B are with gender interactions

|  | **(1a)** | **(2b)** | **(3a)** | **(4b)** | **(5a)** | **(6b)** | **(7)** | **(8)** | **(9)** | **(10)** |
| --- | --- | --- | --- | --- | --- | --- | --- | --- | --- | --- |
|  | **Participation** | | **Voluntary/ charity work** | | **Educational/ training course** | | **Sport, social, or other kind of club** | | **Political/community-related organization** | |
| Widowhood period: 2+ years before (ref.) | | | | | | | | | | |
| 1 year before | -0.02^*^ | -0.03 | -0.02^**^ | -0.01 | 0.03^**^ | 0.03^***^ | -0.01 | -0.03 | -0.0 | -0.01 |
| Year of widowhood | -0.03^**^ | -0.03 | -0.02^*^ | -0.03 | 0.00 | 0.03^**^ | -0.00 | -0.0 | 0.01 | 0.00 |
| 1+ years after | 0.00 | 0.01 | -0.01 | -0.00 | 0.01 | 0.03^**^ | 0.02^*^ | 0.01 | 0.01^*^ | -0.00 |
| 2+ years after | 0.02^**^ | -0.03 | 0.00 | -0.03^*^ | 0.02^***^ | 0.03^***^ | 0.04^***^ | -0.02 | 0.01 | -0.01 |
| Period*gender |  |  |  |  |  |  |  |  |  |  |
| 1 year before # Female |  | 0.01 |  | -0.01 |  | -0.03^**^ |  | 0.03 |  | 0.01 |
| Year of widowhood # Female |  | -0.00 |  | 0.01 |  | -0.03^**^ |  | 0.01 |  | 0.01 |
| 1+ years after # Female |  | -0.01 |  | -0.01 |  | -0.03^**^ |  | 0.02 |  | 0.01 |
| 2+ years after # Female |  | 0.07^***^ |  | 0.03^**^ |  | -0.01 |  | 0.08^***^ |  | 0.02 |
| Observations | 167,328 | 167,328 | 167,328 | 167,328 | 167,328 | 167,328 | 167,328 | 167,328 | 167,328 | 167,328 |

^*^ *p* < 0.10, ^**^ *p* < 0.05, ^***^ *p* < 0.01

**Supplementary Table 5.** Baseline characteristics of people who were already widowed the first time they entered the survey

|  | **Widowers** | **Widows** |
| --- | --- | --- |
| *N* | 2,568 | 11,729 |
| Age | 73.56 (10.15) | 73.01 (10.22)** |
| Health: Fair/poor | 49.9% | 42.4%*** |
| Retired | 83.5% | 71.6% *** |
| How satisfied with life | 7.03 (2.12) | 6.86 (2.18)*** |
| **Social network** |  |  |
| — Network size | 2.16 (1.43) | 2.44 (1.46)*** |
| — Friends | 0.45 (0.85) | 0.48 (0.86) |
| — Children | 1.11 (0.94) | 1.28 (0.97)*** |
| — Siblings | 0.29 (0.64) | 0.29 (0.58) |
| — Others | 0.17 (0.50) | 0.19 (0.50) |
| — Satisfaction with the Network | 8.48 (1.92) | 8.88 (1.63)*** |
| — Close Mean Network | 3.07 (0.70) | 3.21 (0.65)*** |
| **Community Participation** |  |  |
| Any participation | 31.1% | 27.4%*** |
| — Voluntary/charity work | 10.6% | 9.7% |
| — Educational course | 4.5% | 5.8%* |
| — Sport or social club | 20.2% | 14.3%*** |
| — Political/community org. | 4.9% | 2.9% *** |
